# Synergistic Enhancement of Corn Insoluble Dietary Fiber via Combined Radiofrequency Heating and Enzymatic Hydrolysis: Fermentability and Short‐Chain Fatty Acid (SCFA) Production

**DOI:** 10.1111/1750-3841.70548

**Published:** 2025-09-27

**Authors:** Victory Igwe, Deandrae Smith, Christian Mensah, Clay Swackhamer

**Affiliations:** ^1^ Department of Food Science Purdue University West Lafayette Indiana USA

## Abstract

This study aims to enhance the fermentability and health benefits of corn insoluble dietary fiber (IDF) from corn gluten meal (CGM) using radiofrequency (RF) heating at 27.12 MHz and enzymatic hydrolysis (EH) with α‐amylase and protease. The objectives are to characterize the structural modifications of IDF, evaluate the effects of RF heating and EH on gut microbiota composition during in vitro fermentation, and analyze short‐chain fatty acid (SCFA) production to determine the fermentability and potential prebiotic effects of treated IDF using fecal microbiota from human donors. A pilot‐scale RF heating system (1.5 kW, 27.12 MHz) was applied to IDF for 40, 50, and 60 min with electrode gaps of 3.81, 5.08, and 6.35 cm. Fiber substrates (RF‐treated and RF + EH‐treated) were anaerobically incubated at 37°C with fecal slurry from three healthy donors. DNA was extracted from fecal samples, and 16S rRNA gene sequencing was performed to analyze microbial diversity and composition. SCFAs (acetate, propionate, butyrate) were quantified using gas chromatography. Microbial analysis revealed that RF + EH treatment enriched propionate‐producing bacteria, particularly Prevotellaceae, and significantly improved fermentability, as evidenced by increased SCFA production. After 6 h, treated fiber yielded 48.73 mM total SCFAs, a 68.54% increase over untreated fiber. By 24 h, total SCFA production reached 62.65 mM, a 40.78% increase compared to the control. These findings indicate that RF + EH treatment enhances IDF bioavailability, promoting gut microbiota fermentation and increasing SCFA production, thereby supporting a balanced microbiome.

## 1 Introduction

Western dietary patterns have led to a persistent “fiber gap,” with most individuals failing to meet the FDA's recommended daily intake of 38 g for men, 25 g for women, and 19 g for children (Quagliani and Felt‐Gunderson 2017; Human Food Program 2024). Dietary fiber, an indigestible carbohydrate, is vital for gastrointestinal health, regulating blood glucose, promoting satiety, and supporting a balanced gut microbiome through fermentation into short‐chain fatty acids (SCFAs) such as acetate, propionate, and butyrate (Mayo Clinic 2023; Harvard T.H. Chan School of Public Health 2023; Slavin 2013; Makki et al. 2018).

Dietary fiber is generally classified as soluble dietary fiber (SDF) or insoluble dietary fiber (IDF). SDF, found in oats, legumes, and fruits, dissolves in water to form a gel that slows digestion, lowers cholesterol, and serves as a prebiotic for beneficial gut bacteria. In contrast, IDF, abundant in whole grains and vegetables, adds bulk to stool and promotes regularity but is less fermentable due to its dense, rigid structure (Elleuch et al. 2011; Slavin 2013; Stephen et al. 2017).

Corn IDF, particularly from corn gluten meal (CGM)—a byproduct of corn wet milling—illustrates these challenges. While CGM is valued for its protein content in animal feed, its IDF is underutilized in human foods due to its rigid matrix, which limits water retention and microbial fermentability. This underutilization represents a missed opportunity for value addition and revenue generation in the food industry.

Enhancing the fermentability of IDF requires physical, chemical, and biological treatments, each with distinct advantages and limitations. Physical methods such as milling and grinding increase surface area and microbial accessibility but can cause nutrient loss due to heat exposure (Elleuch et al. 2011). Extrusion, which combines heat and mechanical shear, improves fermentability but is energy‐intensive and degrades heat‐sensitive nutrients (Stephen et al. 2017). Steam explosion and autoclaving also disrupt fiber structure but may result in significant nutrient loss (Shukla and Cheryan 2001; Niero et al. 2023). Chemical treatments, including acid or base hydrolysis, can modify fiber solubility and bioactivity but may generate undesirable byproducts (Seth et al. 2015; Dell'Olio et al. 2024). Oxidative treatments improve fermentability but can produce harmful oxidation products (Elleuch et al. 2011). Biological approaches, such as enzymatic hydrolysis (EH), use enzymes like cellulase and xylanase to break down fiber, enhancing water‐holding capacity and solubility (Qi et al. 2023). Although EH is eco‐friendly and specific, it is costly and difficult to scale. Microbial fermentation can further enhance fermentability but requires precise control to ensure consistency (Makki et al. 2018).

Combining radiofrequency (RF) heating with EH offers a promising strategy for IDF modification. RF heating, operating at frequencies such as 13.56, 27.12, and 40.68 MHz, generates heat by interacting with polar molecules, ensuring rapid and uniform temperature distribution (Marra et al. 2009). Unlike conventional thermal methods, RF heating targets fiber structures efficiently with minimal thermal degradation, making it an energy‐efficient pretreatment for EH (Piyasena et al. 2003; Marra et al. 2009; Wang, Wang, et al. 2022). RF heating disrupts hydrogen bonds in cellulose, hemicellulose, and lignin, reducing fiber rigidity and enhancing enzymatic accessibility. EH then further breaks down fiber components, exposing hydrophilic groups that increase solubility and microbial accessibility (Qi et al. 2023). While EH alone is eco‐friendly and specific, its high cost and scalability issues are barriers. RF pretreatment can improve EH efficiency by reducing enzyme requirements and processing time, thereby optimizing fiber functionality for high‐fiber foods, nutraceuticals, and prebiotic formulations.

The novelty of the RF/EH approach is its synergistic application of targeted, energy‐efficient RF pretreatment, followed by EH. Unlike conventional physical or thermal methods, which often cause nutrient loss or require high energy input, RF heating delivers rapid, uniform energy that preserves heat‐sensitive nutrients and efficiently disrupts fiber structure (Marra et al. 2009; Piyasena et al. 2003; Wang, Tang, et al. 2022). This process avoids the drawbacks of chemical and oxidative treatments, such as byproduct formation and safety concerns (Seth et al. 2015; Elleuch et al. 2011). By making fiber more amenable to enzymatic attack, the RF/EH approach reduces enzyme requirements and processing time, resulting in enhanced solubility, improved fermentability, and increased SCFA production (Wang, Tang, et al. 2022; Qi et al. 2023).

In summary, the RF/EH method represents a novel, scalable, and sustainable solution for converting underutilized corn byproducts into functional, fermentable dietary fibers, addressing the fiber gap while supporting gut health and providing new opportunities for value addition in the food industry (Marra et al. 2009; Wang, Tang, et al. 2022; Qi et al. 2023).

## 2 Objectives

This study aims to enhance the fermentability and health benefits of corn IDF from CGM by applying RF heating at 27.12 MHz, followed by EH with α‐amylase and protease. The specific objectives are to:

1. Characterize structural modifications—Investigate the effects of RF + EH on the fiber composition, particle morphology, and molecular interactions of corn IDF, assessing changes that may enhance its bioavailability and fermentability.
2. Evaluate microbial diversity—Examine how RF + EH influences gut microbiota composition during in vitro fermentation, focusing on bacterial communities involved in SCFA production.
3. Analyze SCFA production—Measure and compare SCFA levels (acetate, propionate, butyrate) produced during fermentation of treated IDF to assess its fermentability and potential prebiotic effects, using fecal microbiota from human donors.

## 3 Materials and Methods

### 3.1 Corn Gluten Meal (CGM)

CGM, a byproduct of corn milling, is an abundant and affordable source of fiber, primarily used in animal feed due to its high protein content, typically priced between $500 and $700 per metric ton (Shukla and Cheryan 2001; Awika 2011). It is also used in plant based protein supplements, biodegradable materials, and as a natural herbicide (Seth et al. 2015). However, its dense, poorly soluble, and minimally fermentable corn fiber limits its application in high‐value food industries that require enhanced hydration, texture, and stability (Elleuch et al. 2011). CGM was chosen for this project due to its abundant fiber content, providing a cost‐effective material to explore potential improvements in functionality. The CGM was procured from Primient (Lafayette, IN, USA) and stored in a dark, hermetically sealed container at room temperature (26°C) before use.

### 3.2 Preparation of Insoluble Dietary Fiber (IDF)

CGM was processed to obtain insoluble dietary fiber (IDF)W fractions following AOAC Method 991.43. Dried CGM (moisture content <5% w.b.) was ground using a Cyclotec 1093 mill (Foss A/S) and passed through a 0.8‐mm mesh screen for uniform particle size. The 0.8‐mm mesh was chosen to optimize extraction efficiency by increasing surface area for enzymatic action while minimizing loss of fine particles during processing (Prosky et al. 1992; Elleuch et al. 2011). Uniform particle size is important for reproducibility and accurate dietary fiber analysis (Prosky et al. 1992).

The ground meal was suspended in water at a 1:10 (w/v) ratio and heated to 90°C. Heat‐stable α‐amylase (4 mL; Sigma A3403) was added, and the mixture was incubated with stirring for 2 h to hydrolyze starch. An additional 4 mL of α‐amylase was then added, and incubation continued for another 4 h to ensure complete starch hydrolysis. This step disrupts intermolecular hydrogen and ester bonds within the fiber matrix.

Following α‐amylase treatment, the suspension was centrifuged at 4000 × *g* for 10 min (Sorvall ST 8R centrifuge). The soluble fraction was removed, and the insoluble residue was resuspended in water (1:10 w/v), cooled to 50°C, and treated with 5 mL protease (Sigma P1236) for 4 h with continuous stirring to break down protein fractions and further loosen ester linkages.

After protease treatment, the suspension was centrifuged again at 4000 × *g* for 10 min. The α‐amylase and protease treatments were repeated on the resulting insoluble fiber fraction to maximize removal of starch and protein. The final IDF was then suspended in water (1:5 w/v), washed with distilled water and 80% ethanol, and freeze‐dried to obtain the purified IDF. A portion of this IDF was set aside for subsequent RF treatment and further EH.

### 3.3 Radiofrequency (RF) Treatments

#### 3.3.1 Radiofrequency (RF) Unit

**FIGURE 1:**
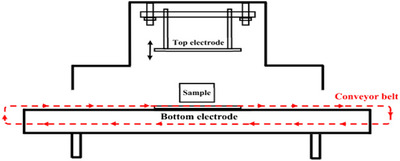
Radiofrequency schematic showing the arrangement of top and bottom electrodes with the sample on a conveyor belt.

A schematic diagram of the pilot‐scale parallel‐plate RF heating system (1.5 kW, 27.12 MHz; Model: SO‐6B, Proctor Strayfield) used to conduct this study is shown in Figure 1. The RF system includes a top and bottom electrode between which the sample is placed. The top electrode has an adjustable product‐to‐emitter gap, while the bottom electrode supports the sample during treatment. A conveyor belt (length: 162.56 cm), outlined in red, continuously moves the samples through the RF treatment zone. The RF unit itself has a width of 60.96 cm, height of 68.58 cm, and length of 162.56 cm, resulting in an internal volume of approximately 679,518.62 cm^3^. These dimensions are critical for understanding energy distribution and treatment uniformity in pilot‐scale applications.

**FIGURE 2:**
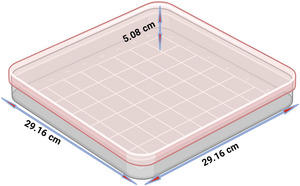
Rectangular high‐density polypropylene (HDPP) tray (29.16 × 29.16 cm).

A product‐to‐emitter gap size of 205 mm was selected for optimized heating without compromising food quality (Smith et al. 2024). The samples were placed in a high‐density polypropylene tray (Figure 2), ensuring proper exposure to the RF field. A fiber optics sensor was connected to ascertain uniform temperature distribution during RF heating.

### 3.4 Radiofrequency (RF) Exposure Parameters

#### 3.4.1 Power Density (*S*) (W/m^2^, W/kg, or W/m^3^)

*Power density (S)* quantifies the amount of energy delivered per unit mass (or, depending on context, per unit area or volume) of a sample during RF exposure. It is commonly calculated using using:

(1) $$S=\frac{P}{m},$$

where *S* is the power density (W/kg); *m* is the sample mass (kg); and *P* is the total power delivered to the sample (W).

#### 3.4.2 Specific Absorption Rate (SAR)

SAR measures the energy absorbed per unit mass of the sample during RF exposure. In this context, it can be calculated as:

(2) $$\mathrm{SAR}=\frac{P_{\mathrm{abs}}}{m},$$

where SAR is the Specific Absorption Rate (W/kg); *P*_abs_ is the power absorbed by the sample (W); *m* is the mass of the sample (kg).

If a gap exists between the product and the RF emitter (e.g., due to sample placement in the tray), the gap volume (V_g_) is:

(3) $$V_{g}=A\times g$$

where A is the cross‐sectional area of the tray (m^2^) and g is the product‐to‐emitter gap size.

#### 3.4.3 Adjusted SAR (SAR_Adjusted_)

SAR_Adjusted_ reflects the cumulative RF energy absorbed by the sample over time. SAR_Adjusted_ values were determined for each treatment duration (40, 50, and 60 min) (Table A1). It was calculated as:

(4) $$\mathrm{SA}R_{\mathrm{Adjusted}}=\mathrm{SAR}\times t,$$

where *t* is the treatment time (s).

#### 3.4.4 Electric Field Intensity (*E*)

Electric Field Intensity (*E*) represents the strength of the electric field within the sample during RF exposure. This formulation integrates the physical dimensions of the product‐to‐emitter gap and electromagnetic properties of the sample to quantify the electric field intensity imparted during RF treatment. The square root relationship highlights the dependence of field strength on applied P and inversely on the product of sample permeability, speed of light, and effective gap area. It was calculated using the following formulas:

(5) $$A_{g}=\frac{V_{g}}{g},$$

(6) $$E=\sqrt{\frac{2P}{\mu \times c\times A_{g}}},$$

where A_g_ is the effective gap cross‐sectional area (m2); V_g_ is the volume of the product‐to‐emitter gap (m_3_); g is the product‐to‐emitter gap size (m); E is the electric field intensity within the sample (V/m); P is the power applied to the sample (W); μ is the sample permeability (H/m); c is the Speed of light in a vacuum (3 × 10^8^ m/s).

#### 3.4.5 Effective Electric Field Intensity (*E*_Eff_)

*E*_Eff_ is the electric field intensity's root mean square (RMS) value. It was calculated as

(7) $$E_{\mathrm{Eff}}=\frac{E}{\sqrt{2}}.$$

Table A1 presents the experimental design conditions for RF‐assisted processing of CGM, focusing on key parameters such as adjusted SAR, *S*, and *E*_Eff_. These values were determined for various RF treatment conditions, including electrode gaps, heating durations, and consistent RF power (1.5 kW). The table below

### 3.5 Temperature Monitoring

Temperature profiles during the RF treatments were monitored using fiber optic sensors (Omega Engineering, Inc., Stamford, CT, USA) to ensure uniform heating and minimize thermal degradation risks.

### 3.6 Enzymatic Hydrolysis (EH) of Radiofrequency (RF) Treated Insoluble Dietary Fiber (IDF)

For further analysis, a subset of the prepared IDF underwent RF treatment. The RF‐treated IDF suspensions (1% w/v) were then subjected to sequential EH as follows:

1. *Protease treatment*: RF‐treated IDF was incubated with protease (P1236, Sigma) at 0.1 U/g IDF at 50°C for 4 h, followed by boiling for 15 min to inactivate the enzyme.
2. *Feruloyl esterase treatment*: The sample was subsequently treated with feruloyl esterase (E‐FAEZCT, Megazyme) at 30 U/g IDF at 50°C for 4 h, followed by boiling for 15 min.

After each enzymatic step, the suspensions were centrifuged at 8000 × *g* for 20 min at 4°C, and the supernatants were collected for analysis. The remaining residues were dried overnight until a constant weight was achieved.

### 3.7 In Vitro Fermentation

The in vitro fermentation was performed based on the method of Lebet et al. (1998) with modifications from Rose et al. (2010). Fiber substrates (RF = radiofrequency‐treated; RF + EH = radiofrequency + enzyme‐treated) were incubated anaerobically at 37°C for 0, 6, and 24 h at 120 rpm with fecal slurry from three healthy donors who were consuming their routine diets and had not taken antibiotics for the last 3–6 months. Two of the donors were male, and one was female, with an age range of 26–38 years (average, 30 years). All donors were within the normal body mass index (BMI) range (18.5–25 kg/m^2^). Fecal samples were collected in sterile plastic tubes, which were immediately sealed, placed on ice, and then transferred into an anaerobic chamber (BactronEZ Anaerobic Chamber; Shel Lab, Cornelius, OR), where in vitro fecal fermentation was performed. All samples were utilized for fermentation within 1 h of collection. Human stool collection and use were approved by the Institutional Review Board at Purdue University (IRB protocol no. 1510016635). Anaerobic carbonate–phosphate buffer was prepared and used to hydrate the substrates overnight. The fecal slurry was added, and gas production was measured at 0, 6, and 24 h. Fermentation was halted with copper sulfate, and pH was measured. Samples were collected for SCFA analysis and frozen at −80°C for DNA extraction. A positive control (fructo‐oligosaccharide [FOS]) and a negative control (blank, no substrate) were included alongside the corn fiber samples. During fermentation, 2 mL of samples were collected at 0, 6, and 24 h for further analysis.

### 3.8 Quantification of Short Chain Fatty Acids (SCFAs)

SCFAs (acetate, propionate, and butyrate) were quantified from fecal slurries as described by Cantu‐Jungles et al. (2021). After centrifugation (13,000 rpm, 10 min), 4 µL of supernatant was injected into an HP 5890 gas chromatograph (GC) equipped with a Nukol capillary column (30 m × 0.25 mm ID, 0.25 µm bonded phase; Supelco, Bellefonte, PA, USA). GC conditions included an injector and detector temperature of 230°C, with an initial oven temperature of 100°C, ramped at 8°C/min to 192°C and held for 3 min. Helium served as the carrier gas at 0.75 mL/min. SCFAs were quantified using a flame ionization detector (FID) and calculated relative to an internal standard (4‐methyl valeric acid) based on peak area.

To ensure consistency across treatments, SCFA concentrations were normalized to the amount of carbohydrate added to each fermentation (50 mg per sample). Final values (mM) were calculated based on SCFA concentration in the sample volume and adjusted per 50 mg of carbohydrate input.

### 3.9 Characterization of Microbial Population

#### 3.9.1 DNA Extraction

DNA extraction from fecal samples collected at 0, 6, and 24 h of in vitro fermentation was performed using the FastDNA Spin Kit for Soil (MP Biomedicals, Solon, OH, USA) following the manufacturer's protocol, with minor modifications as described by Cantu‐Jungles et al. (2021). Briefly, 300 µL of fecal slurry was used for each extraction. The extracted DNA was purified by alcohol precipitation, diluted to a 1–50 ng/µL concentration range, and placed in 96‐well plates. DNA concentration was measured using a NanoDrop 2000c spectrophotometer (Thermo Fisher Scientific Inc., Pittsburgh, PA, USA). The samples were then sent to the Research Bioinformatics Core at Rush University Medical Center (Chicago, IL, USA) for amplicon sequencing of the V4 region of the 16S rRNA gene. Sequencing was performed using a dual PCR strategy. The first PCR amplified genomic DNA with universal primers containing common sequences (CS1_515F and CS2_806R), followed by a second PCR using primers with Illumina sequencing adapters and sample‐specific barcodes. PCR reactions were conducted using AccuPrime SuperMix II (Life Technologies) in 20‐µL volumes for the first stage and 10‐µL volumes for the second stage. The PCR conditions were as follows: initial denaturation at 95°C for 5 min, followed by 28 cycles for stage 1 and eight cycles for stage 2, with denaturation at 95°C, annealing at 55–60°C, and elongation at 68°C. PCR products were verified by agarose gel electrophoresis, pooled, and purified using AMPure XP Beads. The purified products were then prepared for Illumina sequencing, and data analysis was conducted to assess microbial diversity and composition.

### 3.10 Microbial Analysis

Processing and analyzing the 16S rRNA gene sequence data were conducted using methods established in previous studies (Johnson et al. 2019). Briefly, raw sequence data were imported into the CLC Genomics Workbench (QIAGEN, Redwood City, CA, USA) for quality trimming (Q20), and reads shorter than 200 bases were removed. More than 30,000 high‐quality reads were generated per sample. The processed data were then analyzed using QIIME2 and the USEARCH CLUSTering algorithm (UCLUST) with a 97% similarity threshold for operational taxonomic unit (OTU) clustering. Sequence annotation was performed against the Greengenes reference database (version 13.8). The subsequent steps included filtering low‐quality sequences, removing chimeric sequences, and clustering high‐quality reads into OTUs. Finally, alpha‐diversity, beta‐diversity, and taxonomic abundance analyses were conducted using the Phyloseq package in R (version 4.3.1) and JMP.

### 3.11 Statistical Analysis

The in vitro fermentation of IDF samples (treated and native) was conducted in triplicate. Results were expressed as means ± standard deviation. For statistical comparisons among different groups, one‐way analysis of variance (ANOVA), followed by Tukey's HSD post hoc test, was employed at a 95% confidence level (*p* < 0.05). Statistical analysis was performed using JMP (JMP Pro 16.0.0, SAS Institute Inc., Cary, NC, USA).

## 4 Results and Discussion

### 4.1 Optimized Radiofrequency (RF) Exposure Parameters

#### 4.1.1 Power Density (*S*)

*S* was maintained at 17,647.06 W/m^2^, aligning with values reported for RF applications such as paddy rice drying, which reached 300,000 W/m^2^ (Smith et al. 2024). Although this value exceeds the typical RF range of 100–1000 W/m^2^ (Jones and Rowley 1997), it optimizes energy distribution without inducing excessive Maillard reactions or breaking fiber structures. Similar studies on biomass pyrolysis and RF/MW heating (Ibitoye et al. 2025; Rattanadecho and Makul 2016b) support the use of higher power densities to prevent thermal degradation and enhance energy efficiency.

#### 4.1.2 Specific Absorption Rate (SAR)

The samples had a surface area of 850 cm^2^ (0.085 m^2^) and a gap volume of 5.08 cm, The bulk density of the samples was measured at 476.19 kg/m^3^, which is within the acceptable range reported in previous research.

SAR was consistently 729.51 W/kg, with an adjusted range from 1750.81 to 2626.22 W/kg. The optimal adjusted SAR of 1750.81 W/kg provided sufficient energy to disrupt IDF structures while avoiding thermal degradation. SAR directly influences the extent of fiber modification and fermentability. SAR values in the range of 500–2000 W/kg are reported to enhance the accessibility of polysaccharide chains and increase soluble fiber content, thereby improving fermentability by gut microbiota (Wang, Tang, et al. 2022; Rattanadecho and Makul 2016b; Dong et al. 2024). Exceeding 2000 W/kg can cause uneven heating and loss of fiber functionality, which may reduce fermentability due to thermal degradation and altered fiber structure (Smith et al. 2024). Thus, maintaining SAR within the optimal range is critical for maximizing the fermentability of corn IDF while preserving its functional properties.

#### 4.1.3 Electric Field Intensity (*E*_Eff_)

*E*_Eff_ ranged from 105.49 to 136.183 kV/m, with smaller electrode gaps producing higher *E*_Eff_ values (Smith et al. 2024). The optimal *E*_Eff_ of 136.183 kV/m was achieved at a 3.81‐cm electrode gap, which maximized energy absorption and uniform heating—both essential for effective modification of IDF structures. Although smaller electrode gaps can further increase *E*_Eff_, they also raise the risk of uneven heating and potential fiber degradation, as shown by decreased functional properties and possible thermal damage. This level was therefore chosen to balance energy input with fiber quality, consistent with experimental results and literature indicating that optimal electrode spacing and field strength are key for efficient and safe energy application in similar processes (Smith et al. 2024; Rattanadecho and Makul 2016a). Similar findings in cross‐linked polyethylene (XLPE) insulation research confirm that electrode gap variations significantly affectsW energy distribution.

#### 4.1.4 Physicochemical Evidence Supporting Radiofrequency (RF) Parameter Optimization

*S*, SAR, and *E*_Eff_ selections were validated by physicochemical evidence demonstrating improved IDF modification and accessibility. Optimized RF preconditioning (*S* = 17,647.06 W/m, SAR = 1750.81 W/kg, *E*_Eff_ = 136.183 kV/m) reduced median particle size by 39% (320.88 to 196.32 µm) and doubled specific surface area (0.0309 to 0.0744 m^2^/g), enhancing enzymatic efficiency. The RF + EH treatment increased SDF by 50% (4.2% vs. 3.6% for EH alone), lowered IDF content to 42.4%, and improved water‐holding capacity (4.96% vs. 3.09% control), while maintaining structural integrity and increasing amorphous regions. SEM and XRD confirmed enhanced porosity and crystallinity, and color analysis (Δ*E* = 14.59) indicated further structural changes. Compared to conventional methods, RF + EH eliminated chemical residues and minimized nutrient loss, offering a scalable, energy‐efficient solution for functional fiber production (Igwe and Smith 2025).

### 4.2 Microbial Diversity and Community Analysis

#### 4.2.1 Alpha Diversity Indices and Microbial Richness

Alpha diversity was assessed using Chao1, Observed Features, Shannon, and Evenness indices (Table A3; Figures A3, A4, A5, A6). Alpha diversity refers to the diversity of species within a particular area, ecosystem, or sample, typically measured by the number of species present (species richness) and the distribution of individuals among those species (evenness). It provides a local‐scale assessment of biodiversity, offering insight into the structural complexity and health of a community.

*Control* (*CTRL*) refers to the baseline control group in which no dietary fiber treatment or modification was applied. This group represents the standard microbial community composition and diversity, serving as a reference point for evaluating the effects of all experimental treatments.

*POOL* refers to a pooled sample group created by combining equal aliquots from all treatment groups. This composite sample provides an average representation of the microbial community across all experimental conditions, allowing for comparison against individual treatments and the control.

The CTRL exhibited the highest species richness (Chao1 = 236, Observed Features = 226), followed by FOS, POOL, and RF + EH, while RF and BLANK showed significantly lower richness (Makki et al. 2018). The Shannon index revealed the highest diversity in CTRL (6.17), RF + EH (6.15), and POOL (6.14), while RF and BLANK exhibited lower diversity, indicating that RF + EH enhances microbial richness (Paturi et al. 2017).

#### 4.2.2 Principal Coordinate Analysis and Microbial Composition Shifts

Principal coordinate analysis (PCoA) showed that RF + EH clustered similarly to CTRL and FOS (Figure A7), suggesting comparable microbial compositions. RF and BLANK formed distinct clusters, highlighting RF + EH's ability to modulate gut microbiota similarly to prebiotic treatments (Sun et al. 2011; Koh et al. 2016). This treatment increased Prevotella abundance, associated with propionate production, favoring gut microbial health. Increased Bacteroidetes proportions further suggest that RF + EH enhances gut function and supports beneficial bacteria growth (Cummings and Macfarlane 1997; Rios‐Covian et al. 2017).

### 4.3 Microbial Community Composition and Gut Health Implications

#### 4.3.1 Phylum‐Level Shifts: Firmicutes and Bacteroidetes

Firmicutes were most abundant in the BLANK treatment (74.59%), while Bacteroidetes were more prevalent in POOL (24.72%) and RF + EH (24.83%) (Table A2; Figure A8), indicating a favorable shift in the gut microbial community (Zhao et al. 2021; Tett et al. 2021). Minor proportions of Proteobacteria and Actinobacteria were observed across all treatments, reflecting a stable and resilient microbial ecosystem (Koh et al. 2016; Rios‐Covian et al. 2017).

#### 4.3.2 Short Chain Fatty Acid (SCFA) Profiles and Gut Health Outcomes

A higher abundance of Firmicutes, as seen in the BLANK and FOS treatments, correlated with increased butyrate production. Butyrate is a key SCFA that supports gut barrier integrity, reduces inflammation, and provides energy to colonocytes (Makki et al. 2018; Tett et al. 2021). In contrast, the RF and RF + EH treatments, characterized by higher Bacteroidetes, favored propionate production. Propionate is associated with improved glucose metabolism, appetite regulation, and a reduced risk of metabolic disease (Rios‐Covian et al. 2017; Nguyen et al. 2021).

A more balanced Firmicutes/Bacteroidetes ratio and a diversified SCFA profile—especially with increased propionate alongside butyrate—suggest enhanced metabolic and gut health benefits. These microbial shifts can help maintain a healthier gut environment, support immune function, and lower the risk of obesity and metabolic disorders (Makki et al. 2018; Tett et al. 2021).

### 4.4 Short Chain Fatty Acid (SCFA) Production and Nutritional Implications

#### 4.4.1 Temporal Short Chain Fatty Acid (SCFA) Production Dynamics

**FIGURE A1:**
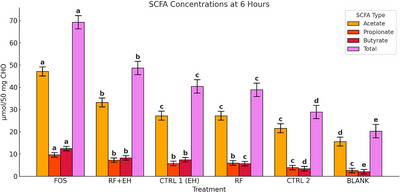
Short‐chain fatty acid (SCFA) production in corn fibers subjected to various treatments (RF, RF + EH, FOS, CTRL 1 (EH), CTRL 2, BLANK) at 6 h. Values are expressed as µmol/50 mg CHO for acetate, propionate, butyrate, and total SCFA. Bars with different letters indicate statistically significant differences (*p* < 0.05). RF = radiofrequency treated; RF + EH = radiofrequency + enzymatic hydrolysis treated; FOS = fructo‐oligosaccharides (+ve control); CTRL 1 (EH) = enzymatic hydrolysis alone; CTRL 2 = native fiber; Blank (−ve control) = no substrate.

**FIGURE A2:**
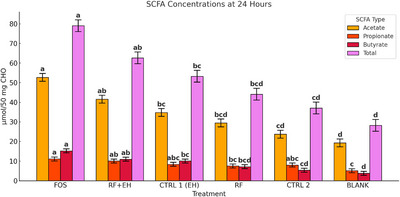
Short‐chain fatty acid (SCFA) production in corn fibers subjected to various treatments (RF, RF + EH, FOS, CTRL 1 (EH), CTRL 2, BLANK) at 24 h. Values are expressed as µmol/50 mg CHO for acetate, propionate, butyrate, and total SCFA. Bars with different letters indicate statistically significant differences (*p* < 0.05). RF = radiofrequency treated; RF + EH = radiofrequency + enzymatic hydrolysis treated; FOS = fructo‐oligosaccharides (+ve control); CTRL 1 (EH) = enzymatic hydrolysis alone; CTRL 2 = native fiber; Blank (−ve control) = no substrate.

**FIGURE A3:**
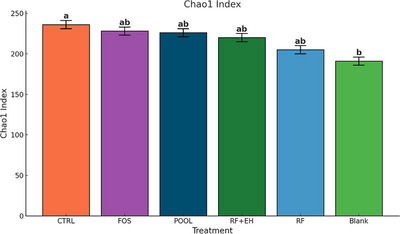
Chao 1 Index of microbial communities across various treatments (RF, RF + EH, FOS, POOL, CTRL, BLANK) at 24 h. Bars with different letters indicate statistically significant differences (*p* < 0.05). RF = radiofrequency treated; RF + EH = radiofrequency + enzymatic hydrolysis treated; FOS = fructo‐oligosaccharides (+ve control); POOL = pooled sample; CTRL = native fiber; Blank (−ve control) = no substrate.

**FIGURE A4:**
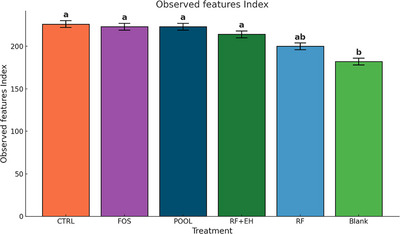
Observed Features Index of microbial communities across various treatments (RF, RF + EH, FOS, POOL, CTRL, BLANK) at 24 h. Bars with different letters indicate statistically significant differences (*p* < 0.05). RF = radiofrequency treated; RF + EH = radiofrequency + enzymatic hydrolysis treated; FOS = fructo‐oligosaccharides (+ve control); POOL = pooled sample; CTRL = native fiber; Blank (−ve control) = no substrate.

**FIGURE A5:**
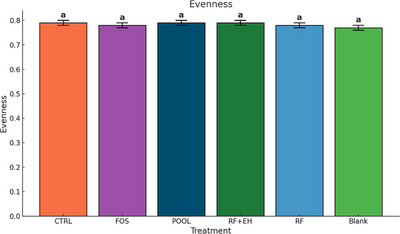
Evenness Index of microbial communities across various treatments (RF, RF + EH, FOS, POOL, CTRL, BLANK) at 24 h. Bars with different letters indicate statistically significant differences (*p* < 0.05). RF = radiofrequency treated; RF + EH = radiofrequency + enzymatic hydrolysis treated; FOS = fructo‐oligosaccharides (+ve control); POOL = pooled sample; CTRL = native fiber; Blank (−ve control) = no substrate.

**FIGURE A6:**
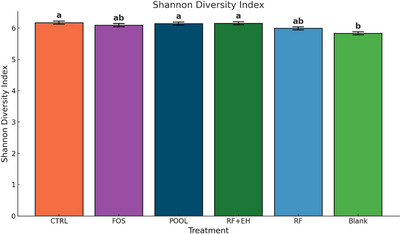
Shannon Index of microbial communities across various treatments (RF, RF + EH, FOS, POOL, CTRL, BLANK) at 24 h. Bars with different letters indicate statistically significant differences (*p* < 0.05). RF = radiofrequency treated; RF + EH = radiofrequency + enzymatic hydrolysis treated; FOS = fructo‐oligosaccharides (+ve control); POOL = pooled sample; CTRL = native fiber; Blank (−ve control) = no substrate.

**FIGURE A7:**
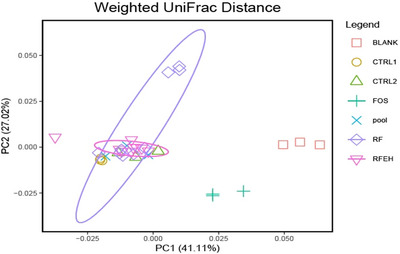
PCoA of microbial communities after 24 h of fermentation. RF = radiofrequency treated; RF + EH = radiofrequency + enzymatic hydrolysis treated; FOS = fructo‐oligosaccharides (+ve control); CTRL 1 (EH) = enzymatic hydrolysis alone; CTRL 2 = native fiber; Blank (−ve control) = no substrate; POOL = pooled samples.

**FIGURE A8:**
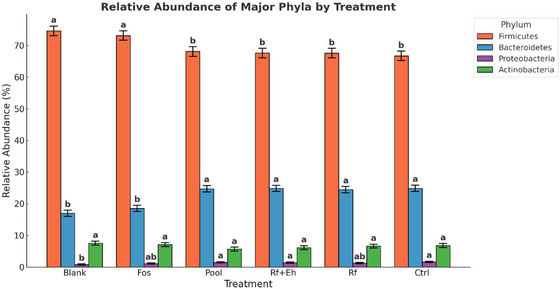
Relative abundance of bacterial phyla across treatments at 24 h. Bars with different letters indicate statistically significant differences (*p* < 0.05). RF = radiofrequency treated; RF + EH = radiofrequency + enzymatic hydrolysis treated; FOS = fructo‐oligosaccharides (+ve control); POOL = pooled sample; CTRL = native fiber; Blank (−ve control) = no substrate.

FOS significantly promoted SCFA production, with acetate, propionate, and butyrate levels all increasing from 6 to 24 h (Figures A1 and A2). RF + EH treatment also enhanced SCFA output, with notable increases in all three major SCFAs over the same period. Importantly, RF + EH increased dietary fiber fermentability by 40.78%, from 43.65 to 62.65 µmol per 50 mg carbohydrate (Table A4; Figures A1 and A2).

#### 4.4.2 Enhanced Fiber Fermentability and Metabolic Benefits

SCFAs are critical for intestinal energy metabolism and overall gut health. They serve as energy sources for colon cells, strengthen the intestinal barrier, reduce inflammation, and support immune function (Du et al. 2024). Enhanced SCFA production, particularly with improved fiber fermentability, is linked to a lower risk of metabolic and inflammatory diseases.

### 4.5 Integrated Health Implications

Together, these findings suggest that both FOS and RF + EH treatments foster beneficial shifts in the gut microbiota and significantly boost SCFA production. These changes are associated with improved gut barrier function, metabolic health, and immune support, highlighting the potential of these treatments to promote overall human health through microbiome modulation and enhanced dietary fiber utilization.

## 5 Conclusion

This study demonstrates the significant impact of different treatments on microbial community composition, diversity, and SCFA production. The optimization of RF exposure parameters, particularly the SAR and *E*_Eff_, has proven to be an effective means of enhancing energy distribution, microbial diversity, and SCFA production without causing thermal degradation.

RF + EH treatment significantly increased the microbial richness and diversity, comparable to the FOS treatment, and promoted beneficial shifts in gut microbiota composition, specifically by increasing the abundance of Bacteroidetes. This shift was associated with enhanced production of SCFAs, such as acetate, propionate, and butyrate, which play crucial roles in gut health and metabolic regulation. The RF + EH treatment, in particular, demonstrated its potential to enhance fiber fermentability and support the growth of beneficial microbial populations, contributing to improved gut health.

The findings also highlight the varying effects of treatments on microbial diversity, with the CTRL group exhibiting the highest diversity, followed by FOS, POOL, and RF + EH treatments. In contrast, BLANK and RF treatments showed lower diversity and SCFA production, suggesting that these treatments may not be as effective in supporting optimal gut microbial activity.

Overall, the study underscores the potential of RF + EH treatment in promoting gut health by enhancing microbial diversity and SCFA production, offering a promising approach for improving dietary fiber fermentation and supporting metabolic health. Further research is needed to explore the long‐term effects of these treatments on gut health and their broader applications in food and nutrition.

Combining RF heating and EH significantly enhances corn IDF fermentability by increasing SCFA production and enriching beneficial gut bacteria. This makes the treated fiber an ideal functional ingredient for promoting digestive health. The scalable, sustainable method offers a cost‐effective solution for developing high‐fiber foods, nutraceuticals, and prebiotic formulations with improved nutritional benefits.

## Author Contributions

**Victory Igwe**: writing – original draft, visualization, formal analysis, data curation, methodology, writing – review and editing. **Deandrae Smith**: conceptualization, funding acquisition, methodology, writing – review and editing, project administration, supervision, resources. **Christain Mensah**: methodology, writing – review and editing. **Clay Swackhamer**: methodology, data curation, writing – review and editing, formal analysis, resources, supervision.

## Conflicts of Interest

The authors declare no conflicts of interest.

## 1

**TABLE A1:** Optimized conditions for RF‐assisted processing of corn gluten meal with adjusted SAR, power density, and effective electric field parameters.

Electrode gap (cm)	*E*_Eff_ (V/m)	Heating duration (min)	SAR_Adjusted_ (kW/kg)
3.81	136,183.00	40	1750.81
5.08	117,937.94	50	2188.52
6.35	105,486.90	60	2626.22

**TABLE A2:** Comparative analysis of dominant phyla across different treatments.

Treatment	Firmicutes (%)	Bacteroidetes (%)	Proteobacteria (%)	Actinobacteria (%)
BLANK	74.59^a^	17.00^b^	0.83^b^	7.58^a^
FOS	73.14^a^	18.54^b^	1.19^ab^	7.12^a^
POOL	68.10^b^	24.72^a^	1.52^a^	5.66^a^
RF + EH	67.62^b^	24.83^a^	1.41^a^	6.15^a^
RF	67.61^b^	24.43^a^	1.32^ab^	6.63^a^
CTRL	66.73^b^	24.84^a^	1.62^a^	6.81^a^

*Note*: Different letters (a, b, c, d, e) indicate significant differences (*p* < 0.05) among treatments at each time point.

**TABLE A3:** Statistical analysis of alpha diversity indices across different treatments.

Treatment	Chao1	Evenness	Observed	Shannon
CTRL	236^a^	0.79^a^	226^a^	6.17^a^
FOS	228^ab^	0.78^a^	223^a^	6.09^ab^
POOL	226^ab^	0.79^a^	223^a^	6.14^a^
RF + EH	220^ab^	0.79^a^	214^a^	6.15^a^
RF	205^ab^	0.78^a^	200^ab^	5.99^ab^
Blank	191^b^	0.77^a^	182^b^	5.83^b^

*Note*: Different letters (a, b, c, d, e) indicate significant differences (*p* < 0.05) among treatments.

**TABLE A4:** Comparative analysis of short‐chain fatty acid (SCFA) production across different treatments.

Treatment	Time (h)	Acetate (mM)	Propionate (mM)	Butyrate (mM)	Total SCFA (mM)
FOS	6	47.15^a^	9.66^a^	12.46^a^	69.27^a^
RF + EH		33.19^b^	7.25^b^	8.29^b^	48.73^b^
CTRL 1 (EH)		27.26^c^	5.78^b^	7.40^b^	40.43^c^
RF		27.21^c^	6.01^b^	5.67^c^	38.90^c^
CTRL 2		21.61^c^	3.91^c^	3.41^d^	28.93^d^
BLANK		15.63^d^	2.60^c^	2.09^e^	20.31^e^
FOS	24	52.72^a^	11.11^a^	15.25^a^	79.08^a^
RF + EH		41.58^ab^	10.08^ab^	10.99^ab^	62.65^ab^
CTRL 1 (EH)		34.83^bc^	8.39^abc^	10.05^bc^	53.27^bc^
RF		29.46^bcd^	7.54^bc^	7.15^bcd^	44.15^bcd^
CTRL 2		23.73^cd^	8.01^abc^	5.36^cd^	37.10^cd^
BLANK		19.28^d^	5.13^c^	3.83^d^	28.23^d^

*Note*: Different letters (a, b, c, d, e) indicate significant differences (*p* < 0.05) among treatments at each time point.
